# Lipoic acid plays a role in scleroderma: insights obtained from scleroderma dermal fibroblasts

**DOI:** 10.1186/s13075-014-0411-6

**Published:** 2014-08-15

**Authors:** Pei-Suen Tsou, Beatrix Balogh, Adam J Pinney, George Zakhem, Ann Lozier, M Asif Amin, William A Stinson, Elena Schiopu, Dinesh Khanna, David A Fox, Alisa E Koch

**Affiliations:** Scleroderma Program, University of Michigan, 300 North Ingalls St. 7C27 NIB, Ann Arbor, MI 48109 USA; University of Michigan Medical School, University of Michigan Medical School, 109 Zina Pitcher Pl, 4388 BSRB, Ann Arbor, MI 48109 USA; Division of Rheumatology, Department of Internal Medicine, University of Michigan Medical School, 300 North Ingalls St. 7C27 NIB, Ann Arbor, MI 48109 USA; Department of Medical Affairs, VA Medical Service, 2215 Fuller Rd., Ann Arbor, MI 48105 USA

## Abstract

**Introduction:**

Systemic sclerosis (SSc) is a connective tissue disease characterized by fibrosis of the skin and organs. Increase in oxidative stress and platelet-derived growth factor receptor (PDGFR) activation promote type I collagen (Col I) production, leading to fibrosis in SSc. Lipoic acid (LA) and its active metabolite dihydrolipoic acid (DHLA) are naturally occurring thiols that act as cofactors and antioxidants and are produced by lipoic acid synthetase (LIAS). Our goals in this study were to examine whether LA and LIAS were deficient in SSc patients and to determine the effect of DHLA on the phenotype of SSc dermal fibroblasts. *N*-acetylcysteine (NAC), a commonly used thiol antioxidant, was included as a comparison.

**Methods:**

Dermal fibroblasts were isolated from healthy subjects and patients with diffuse cutaneous SSc. Matrix metalloproteinase (MMPs), tissue inhibitors of MMPs (TIMP), plasminogen activator inhibitor 1 (PAI-1) and LIAS were measured by enzyme-linked immunosorbent assay. The expression of Col I was measured by immunofluorescence, hydroxyproline assay and quantitative PCR. PDGFR phosphorylation and α-smooth muscle actin (αSMA) were measured by Western blotting. Student’s *t*-tests were performed for statistical analysis, and *P*-values less than 0.05 with two-tailed analysis were considered statistically significant.

**Results:**

The expression of LA and LIAS in SSc dermal fibroblasts was lower than normal fibroblasts; however, LIAS was significantly higher in SSc plasma and appeared to be released from monocytes. DHLA lowered cellular oxidative stress and decreased PDGFR phosphorylation, Col I, PAI-1 and αSMA expression in SSc dermal fibroblasts. It also restored the activities of phosphatases that inactivated the PDGFR. SSc fibroblasts produced lower levels of MMP-1 and MMP-3, and DHLA increased them. In contrast, TIMP-1 levels were higher in SSc, but DHLA had a minimal effect. Both DHLA and NAC increased MMP-1 activity when SSc cells were stimulated with PDGF. In general, DHLA showed better efficacy than NAC in most cases.

**Conclusions:**

DHLA acts not only as an antioxidant but also as an antifibrotic because it has the ability to reverse the profibrotic phenotype of SSc dermal fibroblasts. Our study suggests that thiol antioxidants, including NAC, LA, or DHLA, could be beneficial for patients with SSc.

**Electronic supplementary material:**

The online version of this article (doi:10.1186/s13075-014-0411-6) contains supplementary material, which is available to authorized users.

## Introduction

The pathogenesis of scleroderma (that is, systemic sclerosis (SSc)) includes impaired immunity, vascular abnormalities and tissue fibrosis. Despite the effort expended to understand the disease, the mechanism underlying clinical manifestations of SSc remains elusive. We and others have reported that oxidative stress plays an important role in SSc pathogenesis [1,2], and researchers in a considerable number of clinical studies have also reported that oxidative stress is involved in SSc [3–6]. Interestingly, SSc sera have the ability to induce reactive oxygen species (ROS) production in endothelial cells and fibroblasts [5]. Allanore *et al*. reported that plasma markers of oxidative stress, such as protein carbonyls, nitrosothiols and malondialdehyde, were significantly higher in patients with SSc than in healthy subjects [3]. In addition, the serum levels of 8-isoprostane, another marker for oxidative stress, were found to be elevated in SSc patients and correlated with the severity of pulmonary fibrosis, renal vascular damage and immunological abnormalities [4].

In addition to the increased markers for oxidative stress, studies of decreased antioxidant defense capacity in SSc patients have been reported [6,7]. Several clinical trials were therefore initiated to assess the efficacy of antioxidants in SSc [3,8–10]. Calcium channel blockers (dihydropyridines such as nifedipine) significantly lowered ROS and increased plasma thiol levels [3]. The antioxidative property of nifedipine derives from its ability to reduce superoxide (O_2_•^−^) production from peripheral blood monocytes through the inhibition of protein kinase C (PKC)–dependent protein phosphorylation and PKC activity [10]. Use of a lipid-lowering agent/antioxidant, probucol, not only reduced low-density lipoprotein oxidation susceptibility but also led to significant improvement in Raynaud’s episodes [8]. However, one trial of an antioxidant mixture together with allopurinol failed to show any clinical benefit or improvement in the ROS profile in SSc patients [9]. The purpose of incorporating of allopurinol in that study was to reduce free radical production by inhibiting xanthine oxidase. Allopurinol also produces O_2_•^−^ [11], however, which might be one of the reasons for the negative study result.

The presence of thiols is an indication of oxidative stress in biological systems, as they are very sensitive to ROS and easily oxidized, and therefore they play a crucial role in maintaining the redox state in cells. We showed previously that the free thiol content in SSc dermal fibroblasts was significantly lower than that in normal (NL) cells [1]. It has also been shown that total plasma thiols in SSc patients were significantly lower than in healthy subjects [3,12]. Glutathione (GSH), the most abundant thiol compound in the body, was significantly lower in erythrocytes and dermal fibroblasts from SSc patients compared to the control population [6,13].

In this study, we focused on another crucial thiol, lipoic acid (LA), and its metabolite dihydrolipoic acid (DHLA), in SSc. LA is produced in small amounts by the body via lipoic acid synthetase (LIAS) and acts as the coenzyme for pyruvate dehydrogenase and α-ketoglutarate dehydrogenase in the mitochondria. Together with its reduced form DHLA, it forms a redox couple, and the two act synergistically as biological antioxidants when given orally. In addition, they are capable of regenerating GSH, vitamin C and vitamin E from their oxidized forms [14,15]. In contrast to *N*-acetylcysteine (NAC), a commonly used thiol antioxidant in numerous studies [1,16–18], the LA-DHLA pair appears to provide more benefits as it has a better antioxidative profile and less toxicity. In this study, we were interested in exploring whether LA and LIAS levels are different among healthy subjects and SSc patients, and, because DHLA has more antioxidant activity than LA, we examined whether DHLA affects the profibrotic phenotype of dermal fibroblasts isolated from patients with diffuse SSc.

## Methods

### Patients

All SSc patients fulfilled the American College of Rheumatology criteria for classification of SSc [19]. Plasma samples were collected from SSc patients and healthy subjects. Subject characteristics are listed in Table 1. Two punch biopsies (4 mm) were taken from the forearms of SSc patients with diffuse cutaneous variants. Normal skin tissue was obtained from healthy volunteers as well as the tissue procurement service provided by the University of Michigan Hospital. Written informed consent was obtained for all subjects, and the study was approved by the University of Michigan Institutional Review Board.

**Table 1:** **SSc patient and healthy volunteer characteristics** ^**a**^

	**SSc**	**Diffuse SSc**	**Limited SSc**	**Healthy subjects**
	**(** ***n*** **= 60)**	**(** ***n*** **= 36)**	**(** ***n*** **= 24)**	**(** ***n*** **= 38)**
Age (yr)	57.0 ± 1.4	55.7 ± 1.8	58.2 ± 2.2	44.3 ± 2.4
Sex	F46/M14	F27/M9	F21/M3	F26/M12
Disease duration (yr)	8.7 ± 1.2	5.4 ± 1.0	13.8 ± 2.2	NA
mRSS	13.4 ± 1.5	18.5 ± 2.0	5.3 ± 0.8	NA
Raynaud’s phenomenon	60	36	24	NA
Early disease^b^	31	24	7	NA
Deceased	1	0	1	NA
Digital ulcers	18	14	4	NA
Teleangectasias	38	21	17	NA
Gastrointestinal disease	53	31	20	NA
ILD	30	21	8	NA
PAH	18	8	9	NA
Renal involvement	5	3	2	NA

^a^ILD, Interstitial lung disease; mRSS = Modified Rodnan skin score (0 to 51); PAH, Pulmonary arterial hypertension; SSc, Scleroderma. ^b^Early disease: Less than 5 years. Data are mean ± SEM or raw numbers.

### Cell culture

Both NL and SSc dermal fibroblasts were isolated from human skin. The tissue was digested using enzyme digestion solution containing 2.4 U/ml dispase, 650 U/ml type II collagenase and 10,000 Dornase U/ml DNase. Dermal fibroblasts were maintained in RPMI 1640 medium with 10% fetal bovine serum (FBS), penicillin and streptomycin. Passages between 4 and 8 were used. Before experiments, NL and SSc dermal fibroblasts were switched to RPMI 1640 medium with 0.1% FBS. When needed, 500 μM DHLA (Santa Cruz Biotechnology, Santa Cruz, CA, USA) was added to the cell culture media. The incubation time for DHLA ranged from 48 to 72 hours before the cells were harvested for oxidative stress detection (48 hours) or α-smooth muscle actin (αSMA)/collagen analysis (72 hours). Monocytes from healthy subjects and SSc patients were isolated using Percoll gradient as previously described [20]. They were plated in six-well plates, and the nonadherent cells were washed off after 2 hours of seeding. The adherent cells were monocytes and cultured in RPMI media in the presence of FBS. Before stimulation, they were switched to RPMI media without FBS. The conditioned media were collected for LIAS analysis.

### Oxidative stress detection

Cellular O_2_•^−^ was measured using dihydroethidium (Invitrogen, Carlsbad, CA, USA). The nuclei were stained using 4′,6-diamidino-2-phenylindole (DAPI; Invitrogen). Fluorescence was detected using an Olympus FV-500 confocal microscope, and photographs were taken at 400× magnification. Cellular peroxynitrite levels were detected using 2′,7′-dichlorodihydrofluorescein diacetate [13]. Both NL and SSc dermal fibroblasts were plated in 96-well plates (5 × 10^4^ cells/well, 200 μl/well) and incubated in RPMI 1640 medium (0.1% FBS), with or without 20 mM NAC or 500 μM DHLA, for 48 hours at 37°C. After the incubation, cells were then treated with 200 μM 2′,7′-dichlorohihydrofluorescein diacetate in phosphate-buffered saline for 1 hour at 37°C. The fluorescence intensity was measured using a fluorescence plate reader, and the excitation and emission wavelengths used were 490 and 533 nm.

### mRNA extraction and quantitative RT-PCR

Total RNA was isolated from dermal fibroblasts using RNeasy Mini RNA isolation kits (QIAGEN, Valencia, CA, USA). cDNA was prepared using Verso cDNA synthesis kits (Thermo Scientific, Asheville, NC, USA). Quantitative PCR was performed using SYBR Green PCR Master Mix (Applied Biosystems, Foster City, CA, USA) with specific primers for type I collagen (Col I), density-enhanced phosphatase 1 (DEP-1), Src homology 2 domain–containing protein tyrosine phosphatase 2 (SHP-2) and β-actin. All samples were run in duplicate using Applied Biosystems Real-Time PCR System and analyzed using 7500 Applied Biosystems software.

### Detection of platelet-derived growth factor receptor phosphorylation

Both NL and SSc dermal fibroblasts were incubated with or without 500 μM DHLA overnight and stimulated with platelet-derived growth factor (PDGF). Cell lysates were obtained, and equal amounts of lysate proteins were incubated with immobilized mouse anti-human phospho- tyrosine monoclonal antibody (Cell Signaling Technology, Danvers, MA, USA) overnight at 4°C. Rabbit anti-human platelet-derived growth factor receptor β (PDGFRβ) antibody (Cell Signaling Technology) was used to probe for phosphorylated PDGFR after SDS-PAGE and Western blotting. The immunoprecipitated, tyrosine-phosphorylated proteins were detected using mouse anti-human phospho-tyrosine antibody (Cell Signaling Technology).

### Immunofluorescence

Cells grown in eight-well chambers were fixed and blocked with FBS before being probed with mouse anti-human Col I monoclonal antibody (Abcam, Cambridge, MA, USA), rabbit anti-human αSMA antibody (Abcam) or rabbit anti-LA antibody (Calbiochem, San Diego, CA, USA). Slides were subsequently incubated with Alexa Fluor 488 donkey anti-mouse antibody or Alexa Fluor 488 donkey anti-rabbit antibody (Molecular Probes/Invitrogen, Eugene, OR, USA). The nuclei were stained with DAPI.

### Western blotting

Equal amounts of cell lysate were loaded onto polyacrylamide gels and separated by SDS-PAGE. The proteins were then transferred onto nitrocellulose membranes via Western blotting. The blots were probed with antibodies to LIAS (Thermo Scientific), αSMA (Abcam) or β-actin (Sigma-Aldrich, St Louis, MO, USA).

### Phosphatase activity assay

Phosphatase activity assays were carried out using the DuoSet Intracellular kits from R&D Systems (Minneapolis, MN, USA). Antibodies that capture both active and inactive protein tyrosine phosphatase 1B (PTP1B), SHP-2 or DEP-1 were immobilized. After unbound proteins were washed away, a synthetic phosphopeptide substrate that was dephosphorylated by active phosphatases was added to generate free phosphate and unphosphorylated peptide. The free phosphate was detected by a sensitive dye-binding assay using malachite green and molybdic acid. The activity of the phosphatase was determined by calculating the rate of phosphate release.

### Enzyme-linked immunosorbent assay

Total matrix metalloproteinase 1 (MMP-1), MMP-3, tissue inhibitor of matrix metalloproteinase 1 (TIMP-1) or plasminogen activator inhibitor 1 (PAI-1) in cell culture medium was measured using enzyme-linked immunosorbent assay (ELISA) kits from R&D Systems. Briefly, goat anti-human primary antibodies were coated on a 96-well plate before samples and standards were added. The sandwich ELISA was completed by adding biotinylated goat anti-human antibodies, followed by streptavidin-horseradish peroxidase and substrate solution. The optical density of each well was measured using an ELISA plate reader. Plasma LIAS levels were measured using an LIAS ELISA kit from Cusabio Biotech Co (Wuhan, China). MMP-9 in cell culture media was detected using a SensoLyte Plus 520 MMP-9 assay kit (AnaSpec, Fremont, CA, USA). Endogenously active MMP-1 activity in culture media was detected using a SensoLyte Plus MMP-1 assay kit from AnaSpec.

### Hydroxyproline measurement

Hydroxyproline content in cell culture media was measured using a hydroxyproline assay kit (Sigma-Aldrich). Culture media were treated with concentrated hydrochloric acid and hydrolyzed at 120°C for 3 hours. Hydroxyproline standards, along with 50 μl of the samples, were transferred to a 96-well plate and placed in a 60°C oven until the wells were dried. Chloramine T/oxidation buffer and subsequently the diluted 4-(dimethylamino)benzaldehyde was then added to the wells and incubated for 90 minutes at 60°C. The absorbance was measured at 560 nm using a plate reader.

### Statistical analysis

The results were expressed as mean ± SEM. To determine the differences between the groups, Student’s *t*-tests were performed. *P*-values less than 0.05 with two-tailed analysis were considered statistically significant.

## Results

### LA and LIAS expression

**Figure 1:**
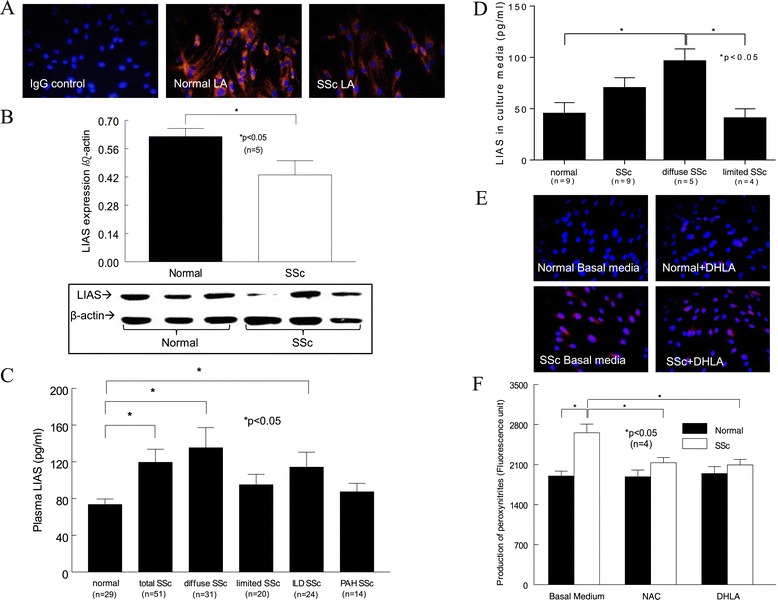
**The levels of lipoic acid, lipoic acid synthetase and oxidative stress in normal and scleroderma fibroblasts and plasma. (A)** Immunostaining of lipoic acid (LA) in normal (NL) and scleroderma (SSc) dermal fibroblasts (*n* = 3 subjects). IgG, Immunoglobulin G. **(B)** The expression of lipoic acid synthetase (LIAS) in dermal fibroblasts was examined by Western blotting (*n* = 6 healthy and 5 SSc subjects). **(C)** Plasma LIAS levels in healthy subjects and SSc patients. ILD, Interstitial lung disease; PAH, Pulmonary arterial hypertension. **(D)** LIAS levels in culture media from monocytes isolated from healthy subjects and SSc patients. **(E)** Cellular superoxide levels were detected with dihydroethidium. These representative images are from five healthy and five SSc subjects. **(F)** The effect of dihydrolipoic acid (DHLA) and *N*-acetylcysteine (NAC) on peroxynitrite levels was measured (*n* = 4 subjects, *P* < 0.05). Results are expressed as mean ± SEM. *P* < 0.05 was considered significant.

Because LA is synthesized in the body, we first examined whether LA expression differed in SSc patients compared to healthy subjects. We found that the cellular expression of LA in SSc dermal fibroblasts was lower compared to NL cells (Figure 1A). In addition, the enzyme that produces LA, LIAS, was significantly lower in SSc dermal fibroblasts (Figure 1B). In contrast, the plasma LIAS levels were significantly elevated in SSc patients, specifically those with diffuse SSc or interstitial lung disease (ILD), compared to healthy subjects (Figure 1C). Patients with limited SSc or pulmonary hypertension showed no difference in LIAS levels. To determine the source of the released LIAS levels detected in plasma, we measured LIAS in conditioned media collected from dermal fibroblasts, dermal endothelial cells and monocytes from both healthy subjects and SSc patients. Only the culture media from monocytes released detectable LIAS levels. Although no statistical significance was reached between NL and SSc groups, there was a significant difference between NL and diffuse SSc (*P* < 0.05) (Figure 1D). Similar to what was seen in the plasma (Figure 1C), monocytes from limited SSc patients did not produce elevated amounts of LIAS (*P* < 0.05 between diffuse and limited SSc) (Figure 1D).

### Oxidative stress

Because LA and its metabolite DHLA can act as antioxidants, we examined their antioxidative capacity in dermal fibroblasts. We found that O_2_•^−^ was significantly higher in SSc dermal fibroblasts (Figure 1E) and that DHLA had no effect. To examine whether DHLA affects other oxidative products, we measured peroxynitrite and found that it was elevated in SSc dermal fibroblasts. NAC and DHLA lowered it significantly (Figure 1F). This suggests that there is increased oxidative stress in SSc dermal fibroblasts and that different thiols affect different forms of oxidative products.

### Phosphorylation of platelet-derived growth factor receptor

**Figure 2:**
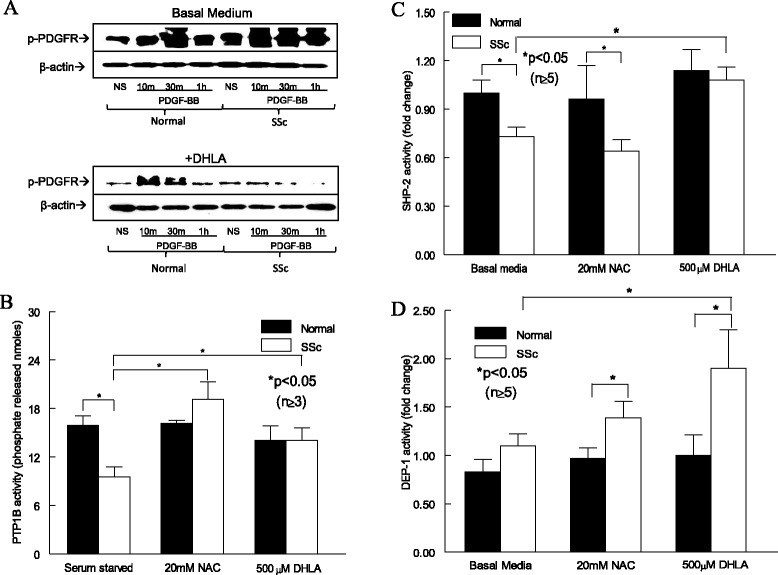
**The effect of dihydrolipoic acid on the platelet-derived growth factor receptor pathway and phosphatases. (A)** Phosphorylated platelet-derived growth factor receptor (p-PDGFR) after PDGF stimulation at various time points (*n* = 3 NL and SSc subjects). NS, Nonstimulated. Enzymatic activities of protein tyrosine phosphatase 1B (PTP1B) **(B)**, Src homology 2 domain–containing protein tyrosine phosphatase 2 (SHP-2) **(C)** and density-enhanced phosphatase 1 (DEP-1) **(D)** in fibroblasts with or without dihydrolipoic acid (DHLA) or *N*-acetylcysteine (NAC). Results are expressed as mean ± SEM. *P* < 0.05 was considered significant.

Because LA was lower in SSc dermal fibroblasts and relieved oxidative stress, we hypothesized that adding DHLA, its active metabolite, to the cells would change their profibrotic phenotype back to normal. We first examined the effect of DHLA on PDGFR activation. In NL dermal fibroblasts, PDGF-stimulated PDGFR phosphorylation (p-PDGFR) was maximal at 30 minutes and decreased significantly at 1 hour (Figure 2A). In contrast, p-PDGFR maximized at 10 minutes and remained phosphorylated at 1 hour in SSc dermal fibroblasts. In the presence of DHLA, p-PDGFR peaked at 10 and 30 minutes for NL fibroblasts. However, PDGF did not induce p-PDGFR in the presence of DHLA in SSc fibroblasts. The time course of p-PDGFR with or without DHLA significantly decreased the extent of p-PDGFR in both NL and SSc. This suggests that the excessive p-PDGFR seen in SSc dermal fibroblasts is due to increased oxidative stress, as a thiol antioxidant could reduce it.

### Phosphatase activities and expression

Because DHLA affects p-PDGFR, we hypothesized that DHLA restores the activities of thiol-sensitive phosphatases by decreasing the oxidative stress in SSc dermal fibroblasts, thereby decreasing p-PDGFR. Because PTP1B, DEP-1 and SHP-2 have been shown to dephosphorylate the PDGFR, we examined their expression and activities with or without DHLA in NL and SSc fibroblasts. SHP-2 mRNA was significantly lower in SSc (Additional file 1: Figure S1), and the presence of the antioxidants increased it, with NAC reaching statistical significance. On the other hand, DEP-1 mRNA was significantly higher in SSc dermal fibroblasts compared to NLs, whereas the thiol antioxidants did not affect the mRNA levels. Interestingly, DEP-1 protein expression was elevated in SSc fibroblasts under basal conditions, and addition of NAC further increased it in both NL and SSc cells (data not shown).

We found that PTP1B was significantly inactivated in SSc dermal fibroblasts (Figure 2B). The addition of thiols significantly restored PTP1B activity. Similarly, SHP-2 activity was significantly lower in SSc dermal fibroblasts (Figure 2C). Whereas addition of NAC had no effect on SHP-2 activity in SSc fibroblasts, DHLA significantly restored it. On the other hand, DEP-1 activity was similar in NL and SSc fibroblasts; however, addition of antioxidants increased DEP-1 activity in SSc dermal fibroblasts (Figure 2D). DHLA was more effective than NAC, as it caused a significant increase in DEP-1 activity in SSc cells compared to that at the basal level. Taken together, these results imply that the increased oxidative stress in SSc inactivates phosphatases responsible for PDGFR dephosphorylation, resulting in increased p-PDGFR. The presence of antioxidants not only eliminates oxidative substances but also restores phosphatase activities, thereby decreasing the extent of p-PDGFR.

### Type I collagen expression

**Figure 3:**
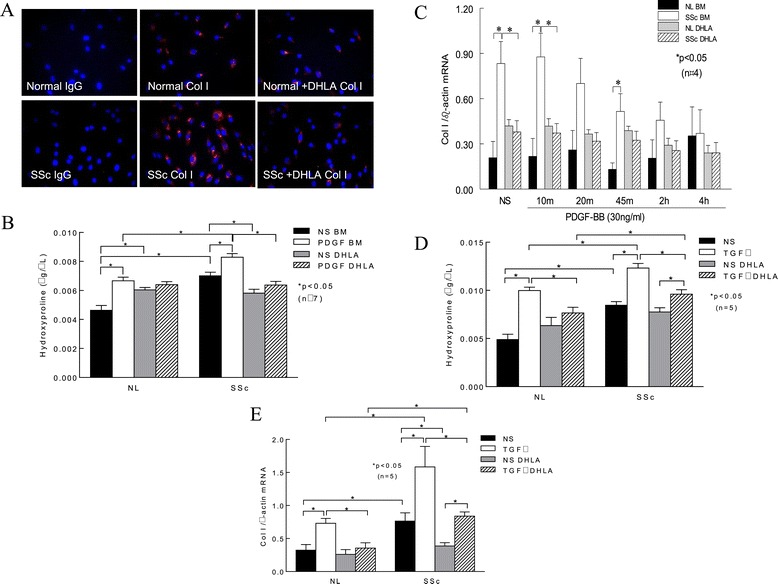
**The expression of type I collagen in fibroblasts. (A)** Immunostaining of type I collagen (Col I) in normal (NL) and scleroderma (SSc) dermal fibroblasts (*n* = 3 NL and 4 SSc subjects). IgG, Immunoglobulin G. **(B)** Hydroxyproline content was measured in cell culture media. As illustrated by the images shown in (A), hydroxyproline levels were elevated in SSc dermal fibroblasts compared to NL cells. Platelet-derived growth factor (PDGF) significantly increased hydroxyproline in both NL and SSc cells. Addition of dihydrolipoic acid (DHLA) significantly reduced hydroxyproline in both NL and SSc cells. **(C)** Col I mRNA levels with or without PDGF stimulation at various time points. **(D)** The effect of transforming growth factor β (TGF**-**β) on hydroxyproline levels was examined. TGF**-**β significantly increased hydroxyproline levels in both NL and SSc dermal fibroblasts, whereas DHLA decreased it. **(E)** TGF**-**β induced Col I mRNA levels in both NL and SSc fibroblasts, and DHLA decreased them significantly. Results are expressed as mean ± SEM. *P* < 0.05 was considered significant. BM, Basal medium; NL, Normal; NS, Nonstimulated.

Because PDGFR activation can lead to excess Col I production, we hypothesized that DHLA can decrease Col I in these cells. As expected, there was more Col I staining in SSc dermal fibroblasts than in NL cells (Figure 3A). In the presence of DHLA, Col I in SSc dermal fibroblasts decreased to levels similar to those observed in NL cells. To further quantify the levels of Col I, we measured hydroxyproline in the cell culture media in the presence or absence of DHLA (Figure 3B). Similar to what is shown in Figure 3A, hydroxyproline was significantly higher in SSc than in NL cells, and addition of DHLA decreased it. Stimulating the cells with PDGF increased hydroxyproline levels significantly in both NL and SSc fibroblasts, whereas DHLA diminished this effect (Figure 3B). We also determined the effect of DHLA at the mRNA level. At basal level and at 10 and 45 minutes after PDGF stimulation, Col I mRNA was significantly higher in SSc fibroblasts (Figure 3C). After 4 hours of PDGF stimulation, Col I mRNA significantly decreased compared to NS. DHLA significantly decreased Col I mRNA levels in SSc fibroblasts at basal levels and 10 minutes after PDGF incubation. These results indicate that, in SSc, enhanced PDGFR activation leads to more Col I synthesis. By acting on scavenging ROS and deactivating the PDGFR, DHLA decreased Col I production.

To further investigate the effect of DHLA on transforming growth factor β (TGF-β), NL and SSc dermal fibroblasts were incubated with 10 ng/ml TGF-β for 48 hours and Col I production was examined by measuring both qPCR and hydroxyproline content. In NL fibroblasts, the addition of TGF-β significantly increased the hydroxyproline content (Figure 3D). In contrast, the presence of DHLA significantly decreased it. Hydroxyproline levels were significantly elevated in conditioned media from SSc dermal fibroblasts compared to NL cells, and they increased significantly after TGF-β incubation. Although the addition of DHLA significantly reduced hydroxyproline levels in SSc cells, the amount of hydroxyproline was still significantly higher compared to that in NL fibroblasts.

The mRNA levels of Col I were also examined after cells were treated with TGF-β (Figure 3E). Col I mRNA levels were significantly elevated in SSc dermal fibroblasts compared to NL cells. TGF-β induced Col I mRNA in both NL and SSc dermal fibroblasts, and the presence of DHLA reduced the levels. These results suggest that DHLA not only affects PDGF-induced Col I production but also acts on TGF-β-mediated fibrotic processes.

### Levels of matrix metalloproteinase, tissue inhibitor of matrix metalloproteinase 1 and plasminogen activator inhibitor 1

**Figure 4:**
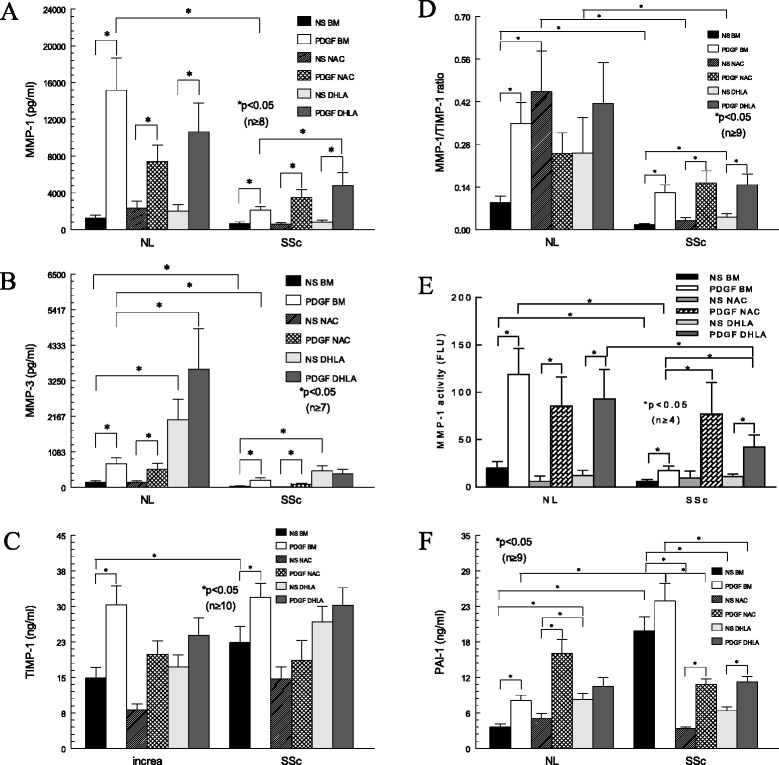
**MMP-1, MMP-3, TIMP-1 and PAI-1 expression; MMP-1/TIMP-1 ratio; and MMP-1 activity in fibroblasts.** **(A)** Matrix metalloproteinase 1 (MMP-1) released into cell culture media after platelet-derived growth factor (PDGF), dihydrolipoic acid (DHLA) and/or *N*-acetylcysteine (NAC) incubation. **(B)** MMP-3 released into cell culture media after PDGF, DHLA and/or NAC incubation. **(C)** Tissue inhibitor of matrix metalloproteinase 1 (TIMP-1) in cell culture media after PDGF, DHLA and/or NAC incubation. **(D)** The MMP-1/TIMP-1 ratio in normal (NL) and scleroderma (SSc) dermal fibroblasts. **(E)** MMP-1 activity was significantly higher in NL dermal fibroblasts than in SSc cells. PDGF significantly increased MMP-1 activity in NL and SSc cells. Both DHLA and NAC appeared to increase MMP-1 activity significantly in SSc dermal fibroblasts when stimulated by PDGF. **(F)** Plasminogen activator inhibitor 1 released from both NL and SSc dermal fibroblasts in the presence of PDGF, DHLA, and/or NAC. Results are expressed as mean ± SEM. *P* < 0.05 was considered significant. BM, Basal medium; NL, Normal; NS, Nonstimulated.

To further examine the effect of thiols on Col I degradation, levels of MMP-1 in cell culture medium were determined (Figure 4A). MMP-1 increased significantly after PDGF stimulation in both NL and SSc fibroblasts. In addition, PDGF-stimulated MMP-1 levels were significantly lower in SSc dermal fibroblasts, which could also contribute to the increased Col I expression seen in these cells. Thiols did not alter MMP-1 significantly in NL cells; however, they did increase MMP-1 in SSc cells stimulated with PDGF. In particular, the presence of DHLA resulted in a significant increase in PDGF-stimulated MMP-1 levels in SSc.

Because MMP-3 converts pro-MMP-1 and pro-MMP-9 into their active forms, we also measured MMP-3 and MMP-9 levels (Figure 4B). Similarly to MMP-1, PDGF stimulated MMP-3 production in both NL and SSc cells. In addition, MMP-3 was significantly lower in SSc fibroblasts under basal conditions. NAC did not appear to have any effect on MMP-3, but DHLA increased MMP-3 significantly in both NL and SSc cells. The lack of MMP-3 to activate MMP-1 may exacerbate the accumulation of Col I in SSc dermal fibroblasts.

MMP-9 was significantly lower in SSc culture medium. Unlike MMP-1 and MMP-3, the antioxidants did not affect MMP-9, nor did PDGF show a consistent effect on its production (data not shown). Similarly, the antioxidants did not affect TIMP-1 levels (Figure 4C). Although TIMP-1 was elevated in SSc dermal fibroblasts, the MMP-1/TIMP-1 ratio was significantly lower in SSc fibroblasts, suggesting a shift that favors a profibrotic phenotype in these cells (Figure 4D). DHLA, but not NAC, increased the ratio significantly, suggesting additional beneficial effects of DHLA on these cells.

To determine whether thiol antioxidants affect MMP-1 activity, we measured endogenous MMP-1 activity in cell culture media. MMP-1 activity was significantly higher in NL cells compared to SSc dermal fibroblasts with or without PDGF stimulation (Figure 4E). Neither NAC nor DHLA had an effect on MMP-1 activity in NL cells. In SSc dermal fibroblasts, Both DHLA and NAC increased MMP-1 activity significantly.

The levels of PAI-1, an inhibitor for urokinase/tissue type plasminogen activator which contributes to Col degradation, were also examined (Figure 4F). PAI-1 released from SSc fibroblasts was significantly higher, whereas the thiols significantly reduced it. Considering this information together, it is possible that DHLA decreases Col I through decreasing PAI-1 and increasing MMPs.

### Expression of α-smooth muscle actin

**Figure 5:**
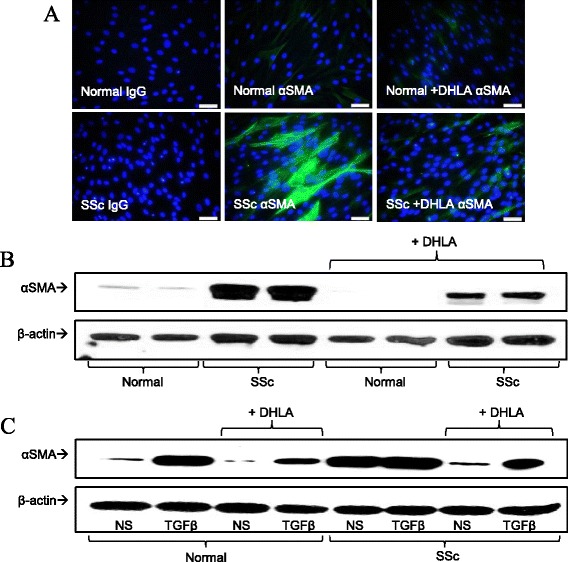
**The expression of α-smooth muscle actin in fibroblasts. (A)** The immunofluorescent staining of α-smooth muscle actin (αSMA) (*n* = 3 subjects). IgG, Immunoglobulin G. **(B)** Western blots (*n* = 4 subjects) show results similar to those in **(A). (C)** Effect of dihydrolipoic acid (DHLA) on transforming growth factor β (TGF-β)–stimulated dermal fibroblasts from healthy and scleroderma (SSc) subjects.

To examine whether DHLA affects the myofibroblast phenotype in SSc, we examined αSMA expression. The expression of αSMA was markedly elevated in SSc dermal fibroblasts, and DHLA reduced it (Figures 5A and B). In addition, we examined the effect of DHLA on TGF-β-stimulated fibroblasts. As shown in Figure 5C, TGF-β induced αSMA expression in both NL and SSc fibroblasts, whereas adding DHLA to the culture decreased its expression.

## Discussion

In this study, we show that the thiol antioxidant DHLA not only decreased cellular peroxynitrites but also reduced p-PDGFR, which is potentially due to its effect on the phosphatases that are involved in this pathway. Col I, αSMA and PAI-1 decreased after DHLA treatment, whereas MMP-1 and MMP-3 increased. In addition, DHLA increased MMP-1 activity. It also decreased the ability of TGF-β to induce Col I and αSMA expression in fibroblasts. The beneficial effect of DHLA might also be due to replenishing the antioxidative capacity in SSc dermal fibroblasts.

NAC is used as an antioxidant, and its effect in SSc has been examined in various studies [1,18,21–23]. Considering the low oral bioavailability of NAC, which could partially explain its limited therapeutic efficacy in clinical trials, the introduction of another thiol antioxidant with a much better absorption profile to treat SSc is an intriguing strategy. LA and DHLA are soluble in both lipid and aqueous environments. Because of this, LA has better bioavailability than NAC; the bioavailabilities of 200 mg of LA and NAC have been estimated to be 38% and 4%, respectively [24,25]. Because DHLA has more reducing activity than LA, it was chosen for our study. We were able to use lower amounts of DHLA and achieve the same or a better effect with it compared with NAC.

We attempted to measure plasma LA and DHLA levels using liquid chromatography-mass spectrometry, but they fell below the detection limit. Instead, we immunostained fibroblasts and found that LA in SSc cells was lower. We should note that the plasma levels of LA and DHLA in healthy subjects have been reported to be approximately 30 ng/ml (that is, 150 nM) and 175 ng/ml (0.84 nM), respectively [26,27], much lower compared to GSH (3.39 ± 1.04 μM) [28], which is the predominant form of cellular thiol. In a separate study, the plasma GSH content in SSc patients was significantly lower than in healthy controls: 170 ± 44 vs. 246 ± 46 μmol/g hemoglobin, respectively [6]. Although DHLA and LA are lower in abundance than GSH, their reduction potential is more negative than that of GSH and its oxidized form (−320 mV vs. −240 mV), suggesting that DHLA can regenerate GSH and maintain the ratio between GSH and it oxidized form GSSG in cells [29,30]. Moreover, owing to their amphipathic nature and smaller molecular sizes compared to GSH, the DHLA-LA pair moves freely in both cytosol and lipid compartments and is readily accessible to more proteins and enzymes that are affected by redox signaling. Therefore, LA and DHLA, whether endogenously or given externally, are indeed important in maintaining cellular function.

We also examined LIAS, a crucial enzyme involved in the LA synthetic pathway. The significantly lower LIAS in SSc fibroblasts might explain the lower LA levels in these cells. However, plasma LIAS was significantly elevated in patients with diffuse SSc. The reason for the discrepancy is not clear. Nonetheless, we were able to determine the source of the LIAS by measuring LIAS in culture media from dermal fibroblasts, endothelial cells and monocytes. It appears that only monocytes from healthy subjects and SSc patients released detectable amounts of LIAS (Figure 1D). Similar to what we saw in the plasma, monocytes from diffuse patients produced elevated amounts of LIAS compared to healthy subjects, whereas those from limited SSc patients did not. Interestingly, we could not detect LIAS from monocytes isolated from patients with rheumatoid arthritis (data not shown). The mechanism of LIAS release in different disease settings appears to be a complicated process and remains to be explored. It is worth noting that 8-isoprostane, an oxidized lipid, was also found to be elevated in plasma obtained from patients with diffuse SSc or ILD (data not shown). It is possible that LIAS is being produced and released from circulating immune cells to counteract the increased oxidative stress observed in SSc patients.

In recent years, the use of LA/DHLA has evolved from antioxidants to antifibrotics. LA was observed to protect against bleomycin-induced lung injury by suppressing ROS and improving the MMP-1/TIMP-1 ratio [31]. Treatment with LA also attenuated cardiac fibrosis in rats [32]. In a diabetic model, LA not only decreased oxidative damage and Col I and αSMA expression in the heart but also increased MMP-2 activity [33]. In addition, LA inhibited the development of thioacetamide-induced liver fibrosis in rats [34]. In a hepatic fibrosis mouse model, LA inhibited the expression of Col I, αSMA and PAI-1 [35]. Our study adds SSc skin fibrosis to the fibrotic diseases that have been shown to be affected by LA/DHLA.

In SSc, fibroblasts differentiate into myofibroblasts, a process characterized by excess production of αSMA. The mechanism of DHLA’s lowering αSMA expression in our study is not known. However, LA treatment has been shown to decrease the expression of TGF-β [33] and affect the redox-sensitive transcription factors activator protein 1 (AP-1) and specificity protein 1 (Sp1) [35]. In addition, it decreased connective tissue growth factor [32]. In hepatic stellate cells, DHLA inhibited TGF-β/PDGF activation through the interruption of ROS-related phosphatidylinositol 3-kinase/protein kinase B (PI3K/AKT) and mitogen-activated protein kinase (MAPK) signaling [34]. Budisavljevic *et al*. had similar results with kidney cells, where mesangial cell transformation into myofibroblasts was completely prevented by LA [36]. These authors showed that LA inhibited the PDGF-activated extracellular signal-regulated kinase 1/2 (ERK1/2) pathway, suggesting that the increased expression of αSMA in cultured mesangial cells could be modulated by redox-sensitive signaling pathways.

Tissue fibrosis occurs when extracellular matrix (ECM) turnover favors production of collagen and other ECM proteins over degradation. Therefore, in this study, in addition to the activation of Col I synthesis (that is, p-PDGFR), we examined the key factors that degrade Col I. MMPs are major proteolytic enzymes involved in degrading and remodeling the ECM. MMP-1 derived from fibroblasts is known to degrade Col I and other collagens. MMP-3 has a broad spectrum of proteolytic activity, including degradation of collagen. Furthermore, MMP-3 is required for maximal activation of pro-MMP-1 and pro-MMP-9. In contrast, TIMP-1 and PAI-1 are natural inhibitors of MMPs and plasmin, thereby inhibiting ECM degradation. In agreement with the results of other studies [37–41], we found that MMP-1, MMP-3 and MMP-9 release by SSc dermal fibroblasts was significantly lower and that TIMP-1 and PAI-1 were elevated. DHLA was able to increase both MMP-1 and MMP-3 levels released from SSc fibroblasts. Although it had minimal effect on TIMP-1, the MMP-1/TIMP-1 ratio increased significantly after DHLA treatment, shifting these SSc cells from a profibrotic state to a relatively antifibrotic state. Moreover, DHLA not only increased the expression of MMP-1 but also increased MMP-1 activity after PDGF stimulation in SSc dermal fibroblasts. This suggests that DHLA can alter pathogenic processes that are important in SSc.

The expression of MMP is regulated by nuclear factor κB (NFκB) [42] and oxidative stress [43,44]; therefore, the presence of antioxidants can alter the expression of MMPs. In our hands, the incubation of thiol antioxidants increased the expression of MMP-1 and MMP-3 (Figure 4). However, MMP-9 expression was not altered by the thiols. It appears that the expression of MMP-9 is regulated by NFκB, AP-1 and the p38 MAPK pathway [45–47] and that LA inhibits MMP-9 expression by inactivating NFκB [48]. The involvement of these other pathways (AP-1 and p38) tightly regulates the MMP-9 expression, and this could be the reason for the ineffectiveness of the thiols used. On the other hand, TIMP-1 expression can be stimulated by a variety of agents, including serum, growth factors, cytokines and viruses [49,50]. Regulation of TIMP-1 expression involves the activation of transcription factors, including AP-1, Sp1, STAT (signal transducer and activator of transcription) and Pea3/Ets families, as well as signaling pathways such as the ERK1/2 pathway [51–53]. Similarly to MMP-9, the complex regulation of TIMP-1 expression makes it difficult to render its expression; hence, in our hands, the use of thiol antioxidants did not affect TIMP-1 levels in NL and SSc dermal fibroblasts.

What could be the possible mechanism by which the thiols affect the expression of the phosphatases examined in this study? Transcription factors, such as NFκB and AP-1, are regulated by the intracellular redox state. They are implicated to be involved in the regulation of expression of a variety of genes [54]. It has been shown that LA and DHLA and NAC inhibit the NFκB pathway [54–56]. NAC also inhibits AP-1 activation [57], whereas the role of LA/DHLA on this pathway is unclear. It is possible that the thiol antioxidants control the expression of phosphatases through these redox-sensitive transcription factors. Moreover, the expression of phosphatases can be affected by oxidative stress [58]. The exact mechanism by which the antioxidants increase SHP-2 and DEP-1 expression requires further investigation.

The PTP family is characterized by their signature motif at their active site, HC(X)_5_R. This site contains an essential cysteine residue that has a low pKa which is susceptible to oxidation. Its ability to be oxidized reversibly acts as a redox regulatory mechanism for receptor tyrosine kinases to control their phosphorylation state. We and others have shown that when excessive oxidative stress is present, the cysteine group is oxidized and the activity is inactivated [1,59,60]. We also reported that PTP1B was oxidized and subsequently inactivated in SSc dermal fibroblasts [1]. This led to prolonged p-PDGFR and increased Col I. Treatment with NAC restored the activity and decreased p-PDGFR and Col I. In this study, DHLA showed a similar effect. In addition to PTP1B, we examined two other thiol-sensitive phosphatases that regulate p-PDGFR. Similar to PTP1B, these phosphatases have a cysteine residue at their active site that is required for their phosphatase activity. As shown in Figure 2, DHLA increased activities of all three phosphatases in SSc fibroblasts. This is superior to NAC, because NAC had no effect on DEP-1 and SHP-2 activities. It has been shown that thiol antioxidants are able to regenerate enzyme − SH groups and restore the phosphatase activities [1,61,62]. Therefore, the mechanism by which DHLA restores the enzyme activity is through reduction of the oxidized cysteine group at the active site. Note that the catalytic activities of these phosphatases are tightly regulated and that subcellular localization can also affect substrate specificity. In other words, the active site may have different susceptibility toward different thiol antioxidants. In addition, the antioxidant capacity of the thiols used in this study is different (the pKa at the thiol group differs), giving them different catalytic activities toward the possible different oxidation products (for example, −SOH, −SO_2_H, −SSR) at the phosphatase active sites [61,63]. Moreover, because the bioavailability of NAC is lower than that of LA/DHLA and LA/DHLA has more desirable structural properties that allow it to move freely in cellular compartments, LA/DHLA could reach a higher concentration intracellularly and hence be more accessible at the enzyme active site compared to NAC. We speculate that these are the reasons why NAC has no effect on SHP-2 activity (Figure 2C), but DHLA does. Nonetheless, the increase in these phosphatase activities provides a potential mechanism for reducing p-PDGFR in SSc dermal fibroblasts.

## Conclusion

To our knowledge, this study is the first to show that LA and LIAS are lower in SSc dermal fibroblasts. In addition, DHLA was able to reverse the profibrotic phenotype of these cells by decreasing p-PDGFR, restoring the activities of phosphatases, lowering PAI-1, and increasing both MMP-1 and MMP-3 expression as well as MMP-1 activity. Moreover, DHLA lowered the expression of αSMA, suggesting that it could reverse the myofibroblast differentiation of SSc dermal fibroblasts. Hence thiol antioxidants, in particular LA or DHLA, could prove to be an effective treatment in SSc.

## Additional file

Additional file 1: Figure S1. Phosphatase expression in NL and SSc dermal fibroblasts. SHP-2 mRNA in SSc dermal fibroblasts was significantly lower than NL (*P* < 0.05, *n* = 6 subjects). On the other hand, DEP-1 mRNA was significantly elevated in SSc dermal fibroblasts compared to NLs (*P* < 0.05, *n* ≥ 5 subjects). The presence of NAC or DHLA did not alter DEP-1 mRNA. NAC significantly increased SHP-2 mRNA in SSc cells, whereas DHLA had no significant effect. Results are expressed as mean ± SE, and *P* < 0.05 was considered significant.

## Abbreviations

Col: Collagen
DHLA: Dihydrolipoic acid
LA: Lipoic acid
LIAS: Lipoic acid synthetase
MMP: Matrix metalloproteinase
NAC: *N*-acetylcysteine
PAI: Plasminogen activator inhibitor
PDGFR: Platelet-derived growth factor receptor
PTP: Protein tyrosine phosphatase
αSMA: α-Smooth muscle actin
SSc: Scleroderma
TIMP: Tissue inhibitor of matrix metalloproteinase
