# Supplementary material for: Lipoic acid plays a role in scleroderma: insights obtained from scleroderma dermal fibroblasts
Source: Arthritis Res Ther. 2014 Aug 15;16(4):4225. doi: 10.1186/s13075-014-0411-6 (PMC4558991; doi:10.1186/s13075-014-0411-6)
Supplement: Additional file 1: Figure S1. — Phosphatase expression in NL and SSc dermal fibroblasts. SHP-2 mRNA in SSc dermal fibroblasts was significantly lower than NL (P < 0.05, n = 6 subjects). On the other hand, DEP-1 mRNA was significantly elevated in SSc dermal fibroblasts compared to NLs (P < 0.05, n ≥ 5 subjects). The presence of NAC or DHLA did not alter DEP-1 mRNA. NAC significantly increased SHP-2 mRNA in SSc cells, whereas DHLA had no significant effect. Results are expressed as mean ± SE, and P < 0.05 was considered significant. [file 13075_2014_411_MOESM1_ESM.docx]

**Supplemental document-**

**S1.** Phosphatase expression in NL and SSc dermal fibroblasts. SHP-2 mRNA in SSc dermal fibroblasts was significantly lower than NL (p<0.05, n=6 subjects). On the other hand, DEP-1 mRNA was significantly elevated in SSc dermal fibroblasts compared to NLs (p<0.05, n≥ 5 subjects). The presence of NAC or DHLA did not alter DEP-1 mRNA. NAC significantly increased SHP-2 mRNA in SSc cells, while DHLA had no significant effect. Results are expressed as mean ± S.E. and p<0.05 was considered significant.
